# Supplementary material for: Differential Item Functioning of the Mini-BESTest Balance Measure: A Rasch Analysis Study
Source: Int J Environ Res Public Health. 2023 Mar 15;20(6):5166. doi: 10.3390/ijerph20065166 (PMC10049698; doi:10.3390/ijerph20065166)
Supplement: Supplementary file 1 [file ijerph-20-05166-s001.zip › ijerph-2251395-supplementary.pdf]

International Journal of
Environmental Research and Public Health

Supplementary Materials S1

Differential Item Functioning for motor disability severity: a control analysis.

The primary analysis showed that completing one or more Mini-BESTest items with an assistive device causes Differential Item Functioning (DIF) in six items of the scale. This DIF is large enough to cause a malfunction of the Mini-BESTest total score. In a sense, the DIF was strong enough to cause a Differential Test Functioning (DTF).

Severely disabled patients are more likely assistive device users. Therefore, it could be suspected that the patients' severity rather than the use of an assistive device is the real cause of DIF. In this scenario, using an assistive device could just be an indicator of severe disability.

A secondary DIF analysis was run to assess this hypothesis.

Patients were split into two classes according to their discharge score on the motor domain of the Functional Independence Measure (FIM). Patients whose motor FIM score was below 80 (i.e. the sample median) were classified as severely disabled. Those who scored ≥ 80 were considered mildly disabled.

Only item 13, "step over obstacles", showed DIF for disability severity. Item 13 was more difficult in severely disabled patients (0.69 logits; SE: 0.12 logits) than mildly disabled ones (0.12 logits; SE: 0.11 logits). This calibration difference was larger than 0.5 logits and statistically significant ($p < 0.001$).

The fact that only one item is affected by DIF for motor disability severity while six items are affected by DIF for assistive devices suggests that using an assistive device instead causes DIF by itself.

Another finding further substantiates this conclusion.

Item 13 also showed DIF for assistive devices. However, the calibration difference between patients with and without assistive devices was much larger than that found for disability (1.98 vs 0.57 logits; see Table 4 in the main manuscript).

Differential Item Functioning for diagnosis: a control analysis.

Four items were affected by DIF for diagnosis, with Item 7 more difficult for peripheral neuropathy than stroke patients and Items 1, 12 and 14 more difficult for stroke (see Table 4 in the main text).

Since the sign of the DIF for item 7 was opposite to items 11, 12 and 14, the measurement artefact caused by items 11, 12 and 14 was partly cancelled out by the one caused by item 7. Therefore, a control analysis was run after removing item 7 to assess the actual size of the measurement artefact caused by the DIF of items 11, 12 and 14.

Copyright: © 2023 by the authors. Licensee MDPI, Basel, Switzerland. This article is an open access article distributed under the terms and conditions of the Creative Commons Attribution (CC BY) license (https://creativecommons.org/licenses/by/4.0/).

Score	Measure Stroke	Measure PNLL	Difference
0	-5.48	-5.63	0.15
1	-4.15	-4.32	0.17
2	-3.28	-3.48	0.20
3	-2.70	-2.93	0.23
4	-2.26	-2.50	0.24
5	-1.88	-2.14	0.26
6	-1.56	-1.82	0.26
7	-1.26	-1.53	0.27
8	-0.99	-1.26	0.27
9	-0.74	-1.00	0.26
10	-0.49	-0.76	0.27
11	-0.26	-0.53	0.27
12	-0.03	-0.30	0.27
13	0.19	-0.08	0.27
14	0.42	0.15	0.27
15	0.65	0.38	0.27
16	0.88	0.61	0.27
17	1.11	0.85	0.26
18	1.36	1.10	0.26
19	1.61	1.36	0.25
20	1.89	1.64	0.25
21	2.19	1.94	0.25
22	2.53	2.28	0.25
23	2.92	2.69	0.23
24	3.44	3.21	0.23
25	4.23	4.01	0.22
26	5.51	5.29	0.22

The table reports the score-to-measure conversion of the Mini-BESTest scale after dropping item 7. Note that the total score (Score) ranges from 0 to 26.

The split-item procedure was applied to items 11, 12 and 14, which are more difficult for stroke patients than patients with peripheral neuropathy of the lower limbs (PNLL). As a result, two calibrations were obtained, one for stroke

patients (Measure Stroke) and the other for patients with peripheral neuropathy (Measure PNLL). The "Difference" column reports the difference: Measure PNLL – Measure Stroke.

Differences ranged between 0.15 and 0.27 logits (median: 0.26 logits). Hence, the measurement artefact caused by DIF of items 11, 12, and 14 (less than 0.5 logits) does not cause much concern.

Disclaimer/Publisher's Note: The statements, opinions and data contained in all publications are solely those of the individual author(s) and contributor(s) and not of MDPI and/or the editor(s). MDPI and/or the editor(s) disclaim responsibility for any injury to people or property resulting from any ideas, methods, instructions or products referred to in the content.
